# Adaptive designs in randomized clinical trials: reanalysis of the HOVON87/NMSG18 multiple myeloma trial

**DOI:** 10.1016/j.eclinm.2025.103605

**Published:** 2025-10-30

**Authors:** Maarten R. Seefat, Niek G. van der Maas, Kazem Nasserinejad, Bronno van der Holt, Anders Waage, Ulf-Henrik Mellqvist, Annette Juul Vangsted, Anna J.T. Smit, Febe Smits, Paula F. Ypma, Niels W.C.J. van de Donk, Hedwig M. Blommestein, Jan J. Cornelissen, David G.J. Cucchi, Sonja Zweegman, Jurjen Versluis

**Affiliations:** a Department of Haematology, Amsterdam UMC, Cancer Centre Amsterdam, the Netherlands; b Department of Haematology, Erasmus MC Cancer Institute, University Medical Centre Rotterdam, Rotterdam, the Netherlands; c HOVON Foundation, Rotterdam, the Netherlands; d Department of Haematology, St Olavs Hospital, Norwegian University of Science and Technology, Trondheim, Norway; e Department of Haematology, Borås Hospital, Borås, Sweden; f Department of Haematology, Rigshospitalet, Copenhagen, Denmark; g Department of Haematology, HagaZiekenhuis, Den Haag, the Netherlands; h Erasmus School of Health Policy and Management, Erasmus University Rotterdam, Rotterdam, the Netherlands; i Department of Internal Medicine, Franciscus Gasthuis & Vlietland, Rotterdam, the Netherlands

**Keywords:** Adaptive trial designs, Multiple myeloma, Randomized clinical trials

## Abstract

**Background:**

Randomized controlled trials are the gold standard to assess clinical efficacy of novel drugs, but improved survival extends follow-up time and challenges their feasibility. Adaptive trial designs, offering earlier outcome assessments, may enhance efficiency and accelerate decision-making. We retrospectively evaluated whether applying adaptive designs to the randomized HOVON87/NMSG18 phase III trial (inclusion between January 2009 and October 2012), which failed to meet its primary endpoint, could have indicated futility earlier.

**Methods:**

We modelled two adaptive trial designs: (1) group sequential and (2) sample size re-estimation. Each design recommends early trial termination for strong efficacy or futility, or continuation if signals are inconclusive. Interim analyses for the group sequential design were defined at 33% and 67% of events required for the final analysis, using O'Brien-Fleming, Pocock, and gamma spending functions to control for false positive or negative results. Sample size re-estimation design was evaluated after 67% of events.

**Findings:**

*Group sequential design*: Hazard ratios (HRs) at both interim analyses were within futility/efficacy boundaries with the more conservative O'Brien-Fleming and gamma spending functions, indicating trial continuation as planned. However, the more aggressive Pocock spending function indicated trial termination at the second interim analysis with a HR of 0.88 exceeding the futility boundary of HR > 0.85. *Sample size re-estimation design*: At 67% of events, the observed HR of 0.86 was in the unfavourable zone, whereas the promising zone required a HR between ≥0.76 and ≤0.83. The observed HR indicated trial continuation without sample size expansion.

**Interpretation:**

This reanalysis of a MM trial suggests that adaptive designs indicate earlier futility detection and thereby facilitate decision-making. Early signals could reduce follow-up time or potentially offer sample size expansion if the effect is promising but not yet conclusive. Our findings highlight the value of implementing adaptive designs to enhance the efficiency and ethical conduct of future RCTs.

**Funding:**

The HOVON87/NMSG18 trial was supported by 10.13039/501100004622Dutch Cancer Society grant 2008-4246, the 10.13039/100008730Norwegian Cancer Society and 10.13039/100006436Celgene.

**Research in context**

**Evidence before this study**

A PubMed search on April 8, 2025, using “adaptive” AND (“phase 3” OR “phase III”) AND “multiple myeloma” identified 80 studies. Notably, while several phase III multiple myeloma trials employed group sequential designs with interim analyses, none implemented sample size re-estimation designs.

**Added value of this study**

This study is the first to retrospectively compare two adaptive trial designs—group sequential (including different spending functions) and sample size re-estimation—in a phase III multiple myeloma trial. By re-analysing the HOVON87/NMSG18 trial data, we highlight the potential for earlier efficacy or futility detection and offer practical insights into how alternative adaptive methods can affect trial conduct.

**Implications of all the available evidence**

Our findings suggest that incorporating more innovative adaptive designs in future trials could shorten follow-up periods, reduce patient exposure to ineffective treatments, and accelerate decision-making. Broad adoption of these approaches may ultimately enhance trial efficiency and improve clinical outcomes. However, these approaches also present practical challenges when implemented in multiple myeloma trials, underlining the need for careful planning and operational consideration.

## Introduction

Randomized controlled trials (RCTs) are the gold standard for evaluating the clinical efficacy of new therapies by assessing the risk-benefit ratio compared to a control group.^1^ Traditionally, RCTs have used fixed designs without interim analyses, or limited early interim analysis to safety and toxicity. In the era of highly effective treatments, the feasibility of RCTs is increasingly challenged by the low frequency of efficacy-related events, often requiring long-term follow-up.^2^

To address these challenges, adaptive trial designs, such as group sequential and sample size re-estimation, can provide timelier results by incorporating interim analyses that assess both futility and efficacy of the primary endpoint.^3^ These designs enable early stopping if an experimental treatment shows early benefit or futility, or allow for increasing the sample size if the expected benefit is promising but not yet conclusive.^3^ A recent reanalysis of the HOVON132 acute myeloid leukaemia trial applied both a group sequential design and Bayesian inference, identifying a very low probability of achieving the expected benefit for the primary endpoint early during trial conduct.^4^ Despite their potential, the use of adaptive designs in haematology remains limited,^5^, ^6^, ^7^ and their added value in MM trials is largely unknown.

We investigated how adaptive trial designs can improve the efficiency of clinical trials by facilitating early detection of efficacy or futility. To this end, we retrospectively applied two adaptive designs, including different spending functions to control for false positive or negative results, to a phase III RCT in newly diagnosed MM patients, which ultimately found no significant difference for the primary endpoint.^8^ In this reanalysis, we evaluated whether and to what extent different adaptive trial designs with interim analyses could have altered the study conduct, and provide a practical overview of how adaptive designs can evolve future RCTs in an era of rapidly expanding therapeutic armamentarium.

## Methods

### Original study design

**Fig. 1:**
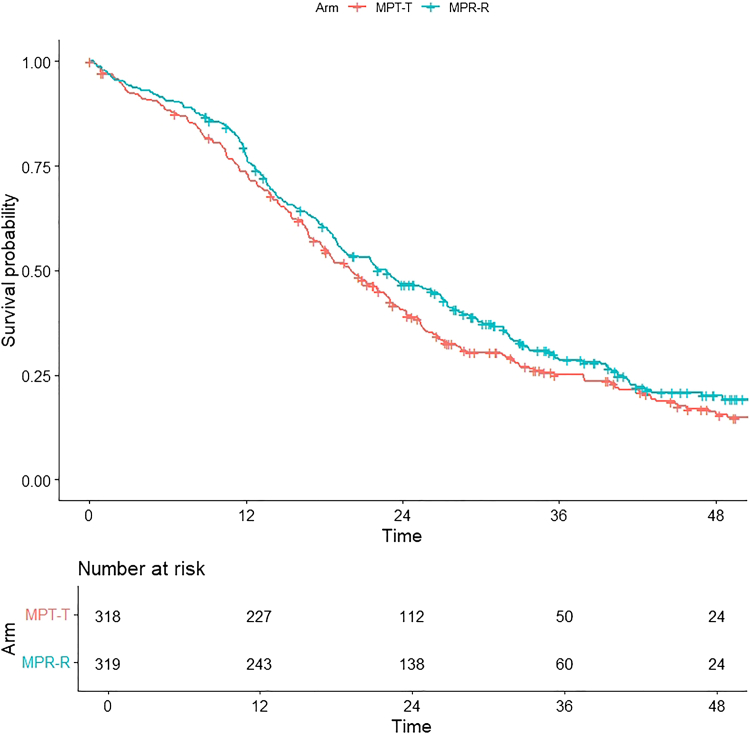
Original Kaplan–Meier curve for progression-free survival (PFS) in the HOVON87/NMSG18 trial. There was no statistically significant difference between both arms, as published previously. Median PFS of 20 (95% Confidence Interval [CI]: 18–23) months for Melphalan-Prednisone-Thalidomide with Thalidomide maintenance (MPT-T) vs 23 (95% CI: 19–27) months for Melphalan-Prednisone-Lenalidomide with Lenalidomide maintenance MPR-R (hazard ratio: 0.87; 95% CI: 0.72–1.04; p = 0.12; adjusted for the International Staging System [ISS]).

The HOVON87/NMSG18 (conducted by the Dutch-Belgium Cooperative Trial Group for Haematology Oncology and the Nordic Myeloma Study Group), trial randomized patients with newly diagnosed MM between melphalan-prednisone with either thalidomide (T) or lenalidomide (R), both during induction and maintenance therapy (MPT-T vs MPR-R). The primary endpoint was PFS. A total of 668 patients with 377 events was required to detect superiority of MPR-R over MPT-T with a hazard ratio (HR) for PFS of 0.714 with 90% power and a two-sided significance level of 5%, with an assumed accrual of 155 patients per year (inclusion was between January 2009 and October 2012). There was one interim analysis for safety and efficacy after 50 PFS events (but without formal stopping rules), which did not indicate alarming findings for either. In the final analysis, 637 patients were eligible and the median PFS was 20 (95% confidence interval [CI]: 18–23) months with MPT-T vs 23 (95% CI: 19–27) months with MPR-R. Using Cox regression, the HR of PFS with MPR-R vs MPT-T was 0.87 (95% CI: 0.72–1.04; p = 0.12) (Fig. 1).^8^

### Adaptive study designs

We retrospectively studied two adaptive designs: a group sequential design and a sample size re-estimation design, both of which incorporated interim analyses for efficacy and futility based on the primary endpoint PFS.

#### Group sequential design

**Fig. 2:**
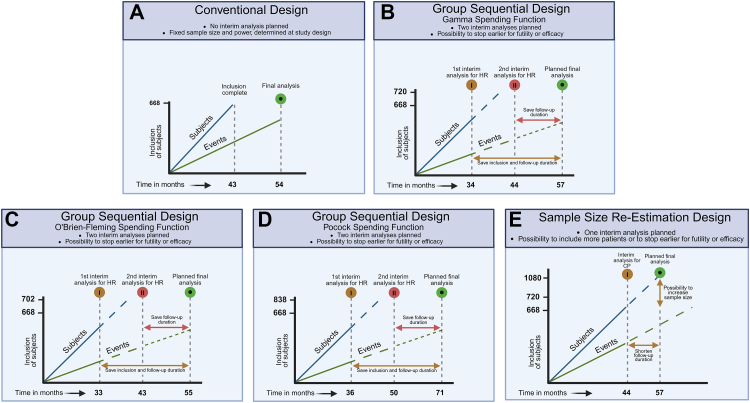
Visualization of different trial designs over time. This figure illustrates the trajectory of subject inclusion alongside the occurrence of events and the decision rules applied in each trial design. Subject inclusion typically progresses at a faster pace, thus interim analyses may have a minimal impact on the rate of inclusion or may not influence it at all. However, there might be a stronger effect on the total amount of events and follow-up time. A. Conventional design: A fixed design without the application of interim analyses with decision rules. B–D. Group sequential design: Two interim analyses are planned, with the possibility to stop earlier for futility or efficacy. B) includes a gamma spending function, C) an O'Brien-Fleming spending function and D) a Pocock spending function. Each function requires slightly more patients than the conventional design, with the most conservative O'Brien-Fleming spending function the least (n = 702) and the most aggressive Pocock spending function the largest sample size (n = 838). The timing of the interim analyses also differ per spending function, as a result of the amount of events needed. E. Sample size re-estimation design: One interim analysis is planned, which not only allows for early stopping for futility or efficacy but also gives the opportunity to include more patients if the results are promising but inconclusive. This design requires slightly more patients than the conventional design.

A group sequential design allows for early stopping of a clinical trial based on predefined, fixed HR boundaries for efficacy and futility at interim analyses. The efficacy and futility boundaries become more narrow at later interim analyses, as more events are observed and the estimate of the endpoint becomes more precise. We investigated two interim analyses at 33% and 67% of total events needed, with different spending functions (see below and Fig. 2B–D).^9^

#### Sample size re-estimation design

In the sample size re-estimation design we applied the promising-zone approach, with predefined HR boundaries for efficacy and futility, similar to the group sequential design. The sample size re-estimation design is based on interim conditional power (CP), which indicates the likelihood of finding a statistically significant difference at the final analysis, based on the data available at the interim analysis. When the CP lies within the promising zone, it implies continuation of the study with sample size re-estimation to increase the study power (Fig. 2E).^10^ Both the unfavourable zone and the favourable zone (above the originally planned study power) imply continuation without adjustments of the sample size, as re-estimation would be unjustified due to a high likelihood of either non-significance (unfavourable zone) or significance (favourable zone) at the preplanned final analysis.^11^ Metha and Pocock have previously established minimum cut-off values for CP based on simulation studies after 50% of events of 41% (CP of 42% at 25% events, and CP of 38% at 75%), which was used as ≤40% CP for unfavourable zone in our analysis at 67% of events. Consequently, the promising zone is defined with CP > 40% and <90%, whereas the favourable zone has a CP ≥ 90%. A single interim analysis at 67% of events^10^ was investigated as this was deemed the optimal compromise between early assessment of efficacy or futility. To facilitate clinical interpretation, we converted the CP in our study to HR values, using default settings in Cytel East, version 6.5. A CP of 40% at the interim analysis corresponded in the HOVON87/NMSG18 trial to an HR of 0.83, and a CP of 90%–0.76. The unfavourable HR interval at the interim analysis is therefore between 0.83 and 0.89, the promising zone interval between 0.76 and 0.83 and the favourable interval between 0.75 and 0.76. Values outside these intervals indicate either futility (HR > 0.89) or efficacy (HR < 0.75). This approach contrasts with group sequential designs, where interim data are only used for evaluating futility or efficacy, but not for sample size re-estimation.^12^

### Family-wise error rate correction

When implementing interim analyses, the risk of a false positive (type I error) or false negative (type II error) result increases.^13^ This can be mitigated by spending functions to keep the overall family-wise error rate within acceptable ranges.^14^ We evaluated three commonly used spending functions. The O'Brien-Fleming and gamma spending functions are designed to control the overall type I error by imposing stringent thresholds for statistical significance at early interim analyses and progressively less stringent thresholds toward the end of the study. These correction methods are considered conservative, because they are stricter at the beginning, thereby minimizing the chance of false positives. For example, if one interim analysis is implemented with a gamma spending function at 50% information, the p-value for early stopping due to efficacy should be below 0.0009.^15^ This approach has been employed in several landmark studies.^15^, ^16^, ^17^ The O'Brien-Fleming is a fixed approach, while the gamma spending function offers flexible adjustment of how stringent the interim analysis thresholds are throughout the study. In addition to the O'Brien-Fleming and gamma spending functions, we evaluated a Pocock spending function, which distributes the correction more evenly over both interim analyses and the final analysis and is therefore considered more aggressive.^18^ Stopping boundaries at interim analyses for all spending functions were generated in Cytel East, version 6.5. The gamma spending function was specified with a parameter of −2 for both the upper and lower bounds and was also applied to the sample size re-estimation design (Supplemental Tables S1 and S2). For all spending functions, a slightly larger sample size is typically required compared to the original study design to maintain the statistical power of the trial while effectively mitigating the risk of false positive or false negative results (Supplemental Table S2).

All calculations were done using Cytel East version 6.5 and R version 4.4.3.

### Trial registration

Data of the HOVON87/NMSG18 trial was used. The original trial registration was registered at www.trialregister.nl (until June 2022) and https://trialsearch.who.int/ as NTR1630.

### Role of the funding source

The HOVON87/NMSG18 trial was supported by Dutch Cancer Society grant 2008-4246, the Norwegian Cancer Society and Celgene. This re-analysis received no funding.

## Results

### Group sequential design—gamma spending function

**Fig. 3:**
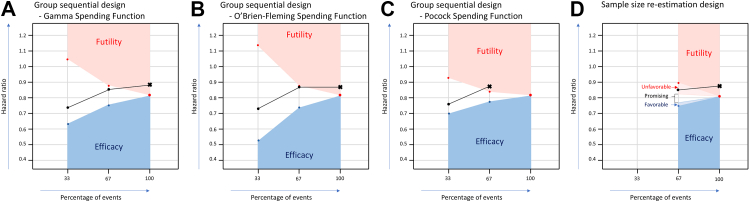
Analysis and decisions of different adaptive designs. A. Group sequential design—Gamma spending function: At the second interim analysis, there was a trend towards futility; however, the evidence was insufficient for stopping the study. In the final analysis, a non-significant difference was observed, with a hazard ratio of 0.88, falling within the futility zone (hazard ratio >0.82). B. Group sequential design—O'Brien-Fleming spending function: Boundaries are most conservative. At the second interim analysis, the observed hazard ratio of 0.87 was just below the futility boundary (hazard ratio >0.88). In the final analysis, a non-significant difference was observed. C. group sequential design—Pocock spending function: Boundaries are most aggressive. At the second interim analysis, the observed hazard ratio of 0.88 crossed the futility boundary (hazard ratio >0.85), indicating early trial discontinuation. D. Sample size re-estimation design: We defined a futility and efficacy zone for earlier stopping at 67% of events. In between these zones, the trial is promising, and the sample size might be re-estimated. At the interim analysis, the hazard ratio was 0.86 (in the unfavourable zone), implying no sample size re-estimation.

When applying a group sequential design with two interim analyses and a gamma spending function, 720 patients and 407 events were required to ensure an overall type I error rate of 5%, instead of 668 patients in the conventional study design. The pre-defined HR boundaries for PFS at the first interim analysis (33% of events) were set to be 1.04 (for futility) and 0.63 (for efficacy). In the first interim analysis, the observed HR was 0.74, being within the pre-defined HR boundaries for PFS. In the second interim analysis (67% of events), the pre-defined HR boundaries were 0.89 (for futility) and 0.75 (for efficacy). The observed HR at the second interim analysis was 0.86, again between the efficacy and futility boundaries indicating continuation of the trial (Table 1 & Fig. 3A). Since the second interim analysis was conducted after accrual was completed, this implicated continuation of follow-up.

**Table 1:** Results and implications for trial conduct of each adaptive design.

	Group sequential design–gamma spending function	Group sequential design–O'Brien-Fleming spending function	Group sequential design–Pocock spending function	Sample size re-estimation design
Sample size	720	702	838	720
Events needed	407	393	503	407
**33% of events Boundaries for:**				
Futilit	HR > 1.04	HR > 1.13	HR > 0.92	NA^a^
Efficacy	HR < 0.63	HR < 0.52	HR < 0.70	
**Analysis**	**HR** = **0.74**	**HR** = **0.73**	HR = 0.76	
**67% of events Boundaries for:**				
Futility	HR > 0.89	HR > 0.88	HR > 0.85	HR > 0.89
Unfavourable				0.83 < HR = <0.89
Promising				0.76 = <HR = <0.83
Favourable				0.75 = <HR < 0.76
Efficacy	HR < 0.75	HR < 0.73	HR < 0.78	HR < 0.75
**Analysis**	**HR** = **0.86**	**HR** = **0.87**	**HR** = **0.88**	**HR** = **0.86**
**100% of events**				
Efficacy	HR < 0.82	HR < 0.82	HR < 0.82	HR < 0.82
**Analysis**	**HR** = **0.88**	**HR** = **0.87**	**NA**	**HR** = **0.88**
**Conclusion**	**Non-significance at final analysis**	**Non-significance at final analysis**	**Stop at second interim analysis**	**Non-significance at final analysis**

NA, Not assessed; HR, Hazard ratio; CP, Conditional power.

The original design of the HOVON87/NMSG18 trial had a sample size of 668 patients, and 377 events were needed.

a No interim analysis planned at 33% of events.

### Group sequential design—O'Brien-Fleming spending function

Application of an O'Brien-Fleming spending function required a sample size of 702 patients and 393 events. At the first interim analysis (33% of events), the pre-defined HR stopping boundaries for PFS were 1.13 for futility and 0.52 for efficacy, wider and more conservative than those of the gamma spending function. The observed HR at this analysis was 0.73, within the boundaries and supporting continuation. At the second interim analysis (67% of events), the futility and efficacy boundaries were 0.88 and 0.73, respectively. The observed HR of 0.87 remained within these limits supporting continuation of the trial.

### Group sequential design—Pocock spending function

A Pocock spending function required a larger sample size of 838 patients with 503 events. The predefined HR boundaries for PFS were more aggressive with futility and efficacy cutoffs of 0.93 and 0.70, respectively, at the first interim analysis (33% of events). The observed HR of 0.76 fell within these limits. At the second interim analysis (67% of events), the stopping boundaries were 0.85 for futility and 0.78 for efficacy. The observed HR of 0.88 crossed the futility boundary, implying trial discontinuation after 335 events, reducing follow-up time.

### Sample size re-estimation design

The sample size re-estimation design included one interim analysis at 67% and would require 720 patients with 407 events. At the interim analysis, the HR was 0.86, which exceeded the predefined unfavourable threshold of 0.83, indicating a low probability of reaching a statistically significant benefit at the final analysis for MPR-R over MPT-T (CP of 14%). The observed HR did not cross the futility boundary of HR 0.89), suggesting trial continuation without a sample size increase based on the interim data. Importantly, all patients would have been included by the time 67% (n = 273) of events had occurred, and a signal for a low probability of trial success would have been identified 9 months before final analysis (Table 1 & Fig. 3B).

## Discussion

We retrospectively applied multiple adaptive designs to the HOVON87/NMSG18 trial for patients with newly diagnosed MM, which randomized MPR-R vs MPT-T and found no difference in PFS at final analysis.^8^ Subsequently, we evaluated results at interim time points and investigated whether the designs could have identified early signals of efficacy or futility. Our present analysis showed that the sample size re-estimation design would not have suggested trial adaptation, whereas futility was only indicated with an aggressive Pocock spending function in the group sequential design. Although the patient inclusion was already completed, this design required less follow-up time to reach a statistically significant signal. These findings suggest that the use of adaptive designs with interim analyses with predefined efficacy and futility thresholds may potentially facilitate earlier decision making, but also present practical challenges when implemented in MM trials.

The sample size re-estimation design used in this analysis has not been applied in phase III MM trials, and the use of group sequential designs has been limited.^5^, ^6^, ^7^ Group sequential designs are suitable for both short- and long-term time to-events and are relatively straightforward to implement (Table 2). In MM studies that adopted this approach, trials generally continued at interim timepoints.^5^, ^6^, ^7^ One such study, the DREAMM-3 trial, ultimately did not meet its primary endpoint, although the median PFS was numerically longer in the experimental arm (11.2 months vs 7.0 months, HR = 1.03, p = 0.56).^6^ Similarly, our reanalysis of the HOVON87/NMSG18 trial using a group sequential design indicated continuation of the trial at both interim analyses with the O'Brien-Fleming and gamma spending functions, despite no benefit upon completion. This reflects the relatively broad and conservative stopping criteria for efficacy and futility of these functions. In contrast, the more aggressive Pocock spending function indicated futility at the second interim analysis. This is consistent with its design, which allocates a greater portion of the overall type I error early in the trial, resulting in a higher probability of stopping the trial early. However, this approach can lead to earlier stopping decisions, while requiring more events later to preserve statistical power. In our analysis, this translated to a relatively later second interim analysis compared to the other methods due to the larger sample size, and might only provide modest time saving in trial conduct. Overall, group sequential designs may be particularly useful when there is substantial uncertainty regarding study parameters for a new therapy, such as event rates, effect size, or potential harm.

**Table 2:** The properties, advantages and disadvantages of group sequential and sample size re-estimation adaptive trial designs.

	Group sequential design	Sample size re-estimation design
Properties	Pre-planned interim analyses to stop the trial for strong evidence for efficacy or futility	Pre-planned interim analyses to stop the trial for strong evidence for efficacy or futility Possibility to expand the sample size to increase study power if the results are promising
Advantages	Few assumptions about design parameters such as the event rate, effect size and harm, therefore more straightforward to implement	May improve chances of study success without compromising integrity, reducing chances of a negative trial while a meaningful effect exists
Disadvantages	Stopping boundaries can be very conservative when O'Brien-Fleming spending functions are used; less conservative shapes such as Pocock are possible but spend more type I error early and require a larger sample size Slightly larger sample size and slightly more events needed Earlier stopping can lead to less long- term data	Slightly larger sample size and slightly more events needed More events/inclusions are needed if results are promising, resulting in a longer trial duration Longer trial durations can lead to capturing a statistically significant outcome with less clinical benefit Earlier stopping can lead to less long-term data
Suitability	Best suitable for both short- and long time-to-events when there are significant uncertainties surrounding the new drug	Best for long time-to-events to detect a meaningful effect size, even if the effect size does not meet the initial study assumptions

In contrast, the criteria in the sample size re-estimation design were set using pre-published CP boundaries, allowing adjustments based on emerging data trends, making it well-suited for trials where events occur slowly, such as in MM.^19^ Moreover, this design is especially useful when patient accrual continues beyond the interim analysis time point that may lead to a sample size re-estimation, as reopening study sites after the completion of initial inclusion can be challenging. In addition, the sample size re-estimation in our study indicated a low probability of trial success 9 months before final analysis, which might be useful to identify trial failure earlier and pivot to alternative strategies, for example if the treatment landscape is evolving rapidly during the trial conduct. By re-estimating the sample size, the chances of study success are improved by reducing the likelihood of a negative trial when a meaningful effect exists, even if the effect size does not meet the initial study assumptions for its primary endpoint (Table 2).^20^ Of note, the currently used cut-offs for effect size in MM trials for investigational therapies are expected to change due to the ESMO-MCBS:H criteria, which define substantial benefit as therapies with a statistically significant HR and an observed 95% confidence interval including 0.65.^21^ Given this context, it would be necessary to restrict the maximum increase in sample size in a re-estimation (e.g., 150% of the original sample size). Such an approach may help avoid finding a statistically significant difference that lacks sufficient clinical relevance and fails to meet the ESMO-MCBS:H criteria.^21^

The ESMO-MCBS:H guidelines emphasize stringent criteria for clinical benefit to reduce unnecessary exposure to less effective treatments, while also quality of life and toxicity are taken into consideration.^21^ Importantly, toxicity was not considered in the efficacy-based interim analyses of these adaptive designs, despite its particular importance in the original HOVON87/NMSG18 trial, which demonstrated a favourable toxicity profile for MPR-R.^8^ Therefore, it is of utmost importance to incorporate toxicity assessments into interim analyses within adaptive designs to ensure balanced evaluation of both efficacy and safety when guiding early decision-making in clinical trials.^4^ These objectives align with super superiority trial designs, which aim to find profound benefits of an experimental arm over a control arm. This is particularly important for high-cost and potentially more toxic treatments and is also relevant if minimal efficacy may not justify market access because the standard of care is already highly effective. However, larger sample sizes are needed, and trial duration may be prolonged because more events need to be observed.

The use of adaptive designs has limitations. First, as survival rates improve in MM,^22^ the future use of adaptive designs is hampered by prolonged events for PFS and OS in MM trials. This may delay early benefit evaluation and reduce the effectiveness of implementing stopping boundaries through adaptive designs. The use of surrogate endpoints like MRD may again expedite the assessment of treatment benefits. Recently, an ODAC endorsed MRD-negativity as an accepted clinical trial endpoint for MM in accelerated approvals, facilitating the use of the latter in adaptive trial designs.^23^^,^^24^ However, final regulatory approval and market authorization still depends on a significant benefit in PFS or OS, as it remains unclear what magnitude of increase in MRD-negative rates is necessary to achieve a clinically meaningful improvement in PFS and OS.^23^, ^24^, ^25^ The FDA has estimated that an odds ratio of 2.12–4.95 for MRD-negative complete response is the minimum likely to translate into a PFS benefit.^23^ Second, although early termination of patient inclusion might not stop trial follow-up, in practice it will often restrict the collection of long-term data, which is crucial for evaluation of the sustained benefits and rare- or late-occurring adverse events of a new drug. This may be addressed by using other sources like long-term follow-up of expanded access programs or real-world data (RWD).^26^ However, ensuring that the quality of non-randomized data approximates that of clinical trial data remains a significant challenge.^27^ Third, adaptive trial designs increase the maximum sample size that must be budgeted for, however they generally reduce the average sample size, because trials may conclude earlier for efficacy or futility compared with a fixed-sample design (Tables 1 and 2, Fig. 2). Fourth, rapid data availability and sufficient data quality may pose a challenge for the investigators, especially when multiple interim analysis are planned. Effective trial management requires the implementation of efficient data cleaning processes that can handle the volume and complexity of trial data without compromising quality. This necessitates close coordination between clinical sites, data management teams and statisticians to ensure that data are consistently monitored, verified and near real-time updated for at least the primary endpoint.

Adaptive designs may optimize development and conduct of phase III trials in the rapidly evolving field of MM where there is a growing need for more efficient and flexible trial methodologies. With regulatory recognition as a valid endpoint, MRD-negativity, in combination with adaptive trial designs, might become a valuable tool for faster assessment of treatment efficacy. This reanalysis of an MM trial suggests that adaptive designs can facilitate earlier decision making with predefined efficacy and futility thresholds. Early indications offer the potential to reduce follow-up time or to increase the sample size if the effect is promising but not yet conclusive; however, this study also highlight several practical challenges associated with their implementation in MM trials. To increase the utilization of adaptive designs, we provide clinical researchers with an overview for designing future clinical trials outlining the properties, advantages, disadvantages and suitability of each design (Table 2).

## Contributors

M.R.S., N.G.v.d.M., K.N., D.G.J.C., S.Z. and J.V. wrote the manuscript and/or made the figures. M.R.S., N.G.v.d.M., K.N. and J.V. analyzed the data. M.R.S., N.G.v.d.M., K.N., J.J.C., D.G.J.C., S.Z. and J.V. developed the methodology. B.v.d.H., A.W., U-H.M., A.J.V., P.F.Y., N.W.C.J.v.d.D., J.J.C., S.Z., conducted the trial and/or collected the data. B.v.d.H., A.W., U-H.M., A.J.V., A.J.T.S., F.S., P.F.Y., N.W.C.J.v.d.D reviewed and edited the manuscript. M.R.S., N.G.v.d.M., K.N., J.V. and B.v.d.H. accessed and verified the data. S.Z. and J.V. decided to submit the manuscript.

## Data sharing statement

Data are available from the corresponding author upon reasonable request. Data will be shared in accordance with institutional and ethical guidelines, ensuring confidentiality and proper use.

## Declaration of interests

BvdH has received honoraria for data safety monitoring board membership from Intergroupe Francophone du Myélome (IFM). NWCJvdD has received research support via his institution from Amgen, Bristol Myers Squibb (BMS), Celgene, Cellectis, Janssen and Novartis, and serves on advisory boards for AbbVie, Adaptive Biotech, Amgen, Bayer, BMS, Celgene, Janssen, Kite Pharma, Merck, Novartis, Pfizer, Roche, Sanofi, Servier and Takeda. HMB has received research grants from The Netherlands Organization for Health Research and Development (ZonMw), Erasmus University medical Centre, Medical Delta, the Dutch Healthcare Institute (ZIN) and the Canada's Drug Agency (CDA), and an advisory board fee from Pfizer, payments were made to the institute, outside the submitted work. DGJC has received payments for lectures for Takeda, and received financial support for travel expenses from Servier and Janssen, all outside the submitted work. SZ: Consulting or Advisory Role: Celgene, Janssen-Cilag, Takeda, Celgene/Bristol Myers Squibb, Sanofi, Oncopeptides (no personal funding); Research Funding: Celgene (funding HOVON 87 study) Janssen, Takeda (both outside the submitted work). JV has received honoraria form AbbVie and Novartis, and consulting/advisory roles for ExCellThera and Rigel.

All others declare no potential conflicts of interest.
