# Supplementary material for: Adaptive designs in randomized clinical trials: reanalysis of the HOVON87/NMSG18 multiple myeloma trial
Source: eClinicalMedicine. 2025 Oct 30;90:103605. doi: 10.1016/j.eclinm.2025.103605 (PMC12613048; doi:10.1016/j.eclinm.2025.103605)
Supplement: Supplemental File [file mmc1.docx]

**Supplemental Table 1.** Different spending functions in the group sequential design

|  | **Early-look spending profile** | **The Gamma spending function** | **Relationship** |
| --- | --- | --- | --- |
| **O’Brien–Fleming** | Extremely conservative early (almost no α spent until late looks) | γ = –4 | As γ → −∞ the HSD spend tends to the Lan–DeMets O’Brien–Fleming curve; γ = –4 is the common practical “O’Brien–Fleming-like” choice. |
| **Pocock** | Equal (or almost equal) α spent at every look | γ = +1 | When γ = 0 the HSD formula reduces to linear spending, which yields nearly constant Z-boundaries—i.e., a Pocock-like design. |
| **Intermediate (e.g., γ = –2)** | Between O’Brien–Fleming and Pocock; moderate early spending | γ = –2 | Gives more flexibility: some early α to allow early stopping, but not as liberal as Pocock. |

**Supplemental Table 2.** Boundaries and cumulative alpha and beta spending by design

|  | | **Group sequential design**  - Gamma spending function | **Group sequential design**  - O’Brien-Fleming spending function | **Group sequential design**  - Pocock spending function | **Sample size re-estimation design** |
| --- | --- | --- | --- | --- | --- |
| **Sample size** | | 720 | 702 | 838 | 720 |
| **Events needed** | | 407 | 393 | 503 | 407 |
| **33% of events** | Dᵢ  cum α(tᵢ)  cum β(tᵢ)  Futility HR  Efficacy HR | 136  0.004  0.015  > 1.04  < 0.63 | 130  0.000  0.004  > 1.13  < 0.52 | 166  0.011  0.045  > 0.92  < 0.70 | NA* |
| **67% of events** | Dᵢ  cum α(tᵢ)  cum β(tᵢ)  Futility HR  Unfavourable HR  Promising HR  Favourable HR  Efficacy HR | 271  0.043  0.011  > 0.89  < 0.75 | 263  0.006  0.044  > 0.88  < 0.73 | 337  0.019  0.076  > 0.85  < 0.78 | 271  0.043  0.011  > 0.89  0.83 < HR ≤ 0.89  0.76 ≤ HR ≤ 0.83  0.75 ≤ HR < 0.76  < 0.75 |
| **100% of events** | Dᵢ  cum α(tᵢ)  cum β(tᵢ)  Efficacy HR | 407  0.025  0.099  < 0.82 | 393  0.025  0.100  < 0.82 | 503  0.025  0.100  < 0.82 | 407  0.025  0.099  < 0.82 |

Abbreviations: NA = Not assessed; Dᵢ = Planned event count; cum α(tᵢ) = Cumulative alpha spent; cum β(tᵢ) = Cumulative beta spent; HR = Hazard ratio; CP = Conditional power.

* No interim analysis planned at 33% of events

The original design of the HOVON87/NMSG18 trial had a sample size of 668 patients, and 377 events were needed
